# Relationship of smoking with current and future social isolation and loneliness: 12-year follow-up of older adults in England

**DOI:** 10.1016/j.lanepe.2021.100302

**Published:** 2022-01-02

**Authors:** Keir EJ Philip, Feifei Bu, Michael I Polkey, Jamie Brown, Andrew Steptoe, Nicholas S Hopkinson, Daisy Fancourt

**Affiliations:** a National Heart and Lung Institute, Imperial College London, London, United Kingdom; b NIHR Imperial Biomedical Research Centre, London, United Kingdom; c Respiratory Medicine, Royal Brompton Business Group, Guys and St Thomas’ NHS Foundation Trust, London, United Kingdom; d Tobacco and Alcohol Research Group, University College London, United Kingdom; e Department of Behavioural Science and Health, University College London, London, United Kingdom

**Keywords:** Smoking, social isolation, loneliness, older people, tobacco

## Abstract

**Background:**

Smoking is often colloquially considered “social”. However, the actual relationship of smoking with current and future social isolation and loneliness is unclear. We therefore examined these relationships over a 12-year follow-up.

**Methods:**

In this cohort study, we used a nationally representative sample of community dwelling adults aged 50 years and over from the English Longitudinal Study of Ageing (N=8780) (45% male, mean(SD) age 67(10) years. We examined associations of self-reported smoking status at baseline assessment, with social isolation (low social contact, social disengagement, domestic isolation), and loneliness (3-item UCLA loneliness scale), measured at baseline, and follow-up at 4, 8 and 12 years, using ordinary least squares regression models.

**Findings:**

At baseline, smokers were more likely to be lonely (coef.=0·111, 95% CI 0·025 – 0·196) and socially isolated than non-smokers, having less frequent social interactions with family and friends (coef.= 0·297, 95%CI 0·148 – 0·446), less frequent engagement with community and cultural activities (coef.= 0·534, 95%CI 0·421 – 0·654), and being more likely to live alone (Odds Ratio =1·400, 95%CI 1·209 – 1·618). Smoking at baseline was associated with larger reductions in social contact (coef.=0·205, 95%CI 0·053 – 0·356, to 0·297, 95%CI 0·140 – 0·455), increases in social disengagement (coef.=0·168, 95%CI 0·066 – 0·270, to coef.=0·197, 95%CI 0·087 – 0·307), and increases in loneliness (coef.=0·105, 95%CI 0·003 – 0·207), at 4-year follow-up) over time. No association was found between smoking and changes in cohabitation status. Findings were independent of all identified confounders, including age, sex, social class and the presence of physical and mental health diagnoses.

**Interpretation:**

Smoking is associated with the development of increasing social isolation and loneliness in older adults, suggesting smoking is detrimental to aspects of psychosocial health. The idea that smoking might be prosocial appears a misconception.

**Funding:**

UK Economic and Social Research Council & Imperial College London.

**Research in context**

**Evidence before this study**

Smoking, social isolation, and loneliness are all related to increased morbidity and mortality. Smoking is often colloquially considered “social”. However, the relationship between smoking and social isolation and loneliness is unclear. We searched MEDLINE and Google Scholar using the keywords “smoking” AND “social isolation” AND/OR “loneliness”, for articles published up to July 1, 2021. Identified research largely focuses on social isolation and loneliness leading to smoking, with very few studies considering the idea that smoking might increase or decrease social isolation and loneliness, with a particular lack of research examining whether smoking is related to changes in social isolation and loneliness over time.

**Added value of this study**

To our knowledge this is the first study to examine these relationships using a nationally representative sample of older people, using data from a prospective cohort study with a 12-year follow-up. Our analysis substantially advances the understanding of this topic, which was highly limited, demonstrating that smoking was associated with increased social isolation and loneliness cross-sectionally and over time, independent of a wide range of demographic and health related factors.

**Implications of all the available evidence**

Smoking is associated with increased social isolation and loneliness in older adults, highlighting an opportunity for intervention. These findings suggest the idea that smoking might be pro-social is a misconception, which should be challenged. Our findings support both interventions that reduce smoking initiation, and those that support smoking cessation.

Alt-text: Unlabelled box

## Introduction

Smoking is often colloquially considered “social”. Proposed mechanisms by which smoking could be perceived as prosocial include the potential to provide a sense of social belonging,^1^ and possibly more relevant in younger people, by facilitating social connections and interactions across genders, helping structure time and space in situations such as parties, and identification with smoking peers.^2^^,^^3^ Indeed, some people describe themselves as ‘social smokers’.^4^ Conversely, many other people find smoking highly antisocial,^5^ and the increasingly widespread recognition of the negative health impacts of smoking, and passive smoking, are thought to be key factors in reducing the perceived social acceptability of smoking in high-income countries.^5^^,^^6^

While the impacts of smoking tobacco on physical health are well established, its impact on psychosocial factors such as social isolation and loneliness are far less well understood. Previous research has identified associations between social isolation, loneliness, and smoking, but has largely focused on the idea that social isolation and loneliness lead to more smoking, due to various reasons including reduced exposure to social pressures and social contexts in which smoking is discouraged.^7^, ^8^, ^9^, ^10^ However, these relationships could be bi-directional: smoking could also lead to increased social isolation and loneliness.^11^ Potential mechanisms include the development of smoking related diseases; the onset of limitations affecting physical mobility that consequently impact on one's ability to interact; changing social norms around smoking, with smoking becoming less socially acceptable due to increased awareness of health impacts;^5^^,^^6^ and smoke-free legislation for public spaces,^12^ which could all impair social participation for smokers.

We hypothesised that overall, smoking would result in increased social isolation and loneliness. Given the lack of research in this area, and the theoretical potential for the converse to be true, we sought to clarify the effect of smoking on both social isolation and loneliness, using longitudinal data over a 12-year period from a prospective cohort of adults aged 50 or over in England.

## Methods

Data were drawn from the English Longitudinal Study of Ageing (ELSA), which is a population based, longitudinal panel study of a nationally representative sample of adults aged 50 years and over, and their partners, living in England.^13^ ELSA is designed to explore social, economic, biological and psychological factors relevant to ageing. The initial ELSA sample was recruited in 2002/2003, and consisted of a random stratified sample of households in England who participated in the Health Survey for England (HSE), which is an annual cross-sectional survey of households.^14^ The core ELSA sample was recruited from people who participated in the HSE in 1998, 1999 and 2001, to enable a sufficiently large nationally representative sample to be created. Participation rate data from the HSE is not provided as the surveys were only used to identify people in the process of developing the nationally representative ELSA sample. Creating wave 1 of ELSA, the overall individual response rate (the total number of eligible individuals within all issued households) was 66%. ELSA participants are followed biennially with data collection termed ‘waves’. Further background information on ELSA is available at https://www.elsa-project.ac.uk/about-elsa. Relevant variables for this analysis were measured in ELSA wave 2 (2004/2005), through wave 4 (2008/2009), wave 6 (2012/2013), and wave 8 (2016/2017). For these analyses, the core ELSA sample (N=8,780) was used to ensure national representativeness. The primary method of data collection in ELSA is computer assisted interviews, completed face-to-face at the participants’ usual place of residence. This was the method by which the data analysed here were collected, except for the mobility impairment covariate which was taken from data collected by a qualified nurse with training in the study protocols during a physical assessment. Study materials and documentation can be accessed here https://www.elsa-project.ac.uk/study-documentation, and further information on sampling and the study more generally is available at https://www.elsa-project.ac.uk/. ELSA received ethical approval from the National Research Ethics Service and all participants provided informed consent.

### Measures

#### Smoking status

Smoking status was measured by the question on ‘whether smokes cigarettes at all nowadays’ at baseline wave (2004-2005). It was categorised as a binary variable: current smokers vs not current smoker (never smokers or ex-smokers). Current smoking at baseline was used, rather than current smoking at each wave, given our hypothesis that accumulated smoking related illness and physical mobility limitations could contribute to relationships between variables of interest. Hence our approach limited the extent individuals with extensive smoking histories could move between categories. It is likely that our ‘not current smokers’ included individuals who quit smoking not long before the baseline data collection was taken, through to those that only smoked for a short time. As clear quantification of lifetime smoking exposure was not available, we decided our approach was the most suitable to address our hypothesis.

#### Social isolation

We used three measures of social isolation as in previous ELSA studies (low social contact, social disengagement, and domestic isolation),^15^ to explore whether smoking is related to different aspects of social isolation. Data for these measures were collected at each wave included in the analysis.

Low social contact was assessed using self-reported frequency of social interactions. This included (a) face to face interaction, (b) telephone conversations, or (c) email or written communication with (1) children, (2) other family members, or (3) friends. One point was given per mode of communication with each group of people that an individual did not have contact with at least once per month. This was summed to provide an overall score from 0 to 9 with higher scores indicating lower social contact (greater social isolation). The index had a Cronbach's alpha of 0·77.

Social disengagement was assessed using self-reported frequency of participation in community group activities (including political parties, trade union or environmental groups, tenant groups, resident groups, neighbourhood watch groups, church or other religious groups, charitable associations, education, arts or music groups or evening classes, social clubs, sports clubs, exercise classes, or any other organisations, clubs or societies), and engagement with community cultural activities (including going to museums, exhibitions, the theatre, concerts, opera or the cinema). Frequency of engagement with these groups and activities was measured as number in the past 12 months and then recoded as never (score of 4), once or twice a year (score of 3), every few months (score of 2), or monthly or more (score of 1). Frequency of cultural activities was as never (score of 4), less than once a year (score of 3), once or twice a year (score of 2), or every few months or more (score of 1). These scores were then summed, providing an overall index of 2–8, with higher scores indicating higher levels of social disengagement. The index had a Cronbach's alpha of 0·76.

Domestic isolation was measured as a binary variable based on whether individuals lived alone or with others.

#### Loneliness

Loneliness was measured using an adapted 3-item version of the UCLA loneliness scale,^16^ which has strong psychometric properties.^17^ Respondents were asked how often they (1) felt that they lacked companionship, (2) felt left out, and (3) felt isolated from the people around them. Responses ranged from hardly ever or never (scored as 1) some of the time (scored as 2) and often (scored as score of 3). The scores for each measure were then summed to give a loneliness score ranging from 3 to 9 where higher scores indicated higher levels of loneliness. Data for this measure were collected at each wave included in the analysis. The index had a Cronbach's alpha of 0·83.

#### Covariates

Potential confounders were identified using directed acyclic graphs and included as covariates. Demographic covariates included age (continuous), sex (male, female), ethnicity (white vs ethnic minority, grouped together due to the low number of ethnic minority groups), educational attainment (no qualifications or NVQ level 1, GCE/O Level, A level or other higher education, and degree or above), and employment status (currently working full- or part-time vs not working). Net non-pension wealth (in quintiles) is a robust indicator of socio-economic resources and living standard in the ELSA population,^18^ so was included as an indicator of socioeconomic status.

Health related covariates included self-reported doctor diagnosis of any of the following: heart failure, heart attack, angina, other heart problems, diabetes, stroke, dementia, arthritis, Parkinson's disease, and chronic lung disease (such as chronic bronchitis and asthma), depression (scores above threshold of 3 on the Centre for Epidemiological Studies scale CES-D),^19^ and presence of a mobility impairment (impairment, no impairment) observed by the interviewer in relation to the timed walk test. Baseline data for covariates were included in the analyses.

### Statistical Analysis

Data were analysed using ordinary least squares regression models to estimate the cross-sectional and longitudinal relationships of baseline smoking status with social isolation measures and loneliness, except for domestic isolation, for which we used binary logistic regression. The term ‘longitudinal relationships’ is used to describe the association of baseline smoking status as the independent variable and social isolation and loneliness variables assessed in subsequent years (4, 8, and 12-year follow-up) as dependent variables. Models were built in steps with covariates entered sequentially. Model 1 adjusted for demographic covariates (age, sex, ethnicity, educational attainment, employment status, and wealth). Model 2 additionally adjusted for health-related covariates (medical diagnoses, depression, and mobility impairment). Fully adjusted longitudinal analyses (Model 3) also adjusted for relevant outcome variable at baseline.

Missing data were imputed using multiple imputation by chained equations using all predictor variables used within the analyses to provide 50 imputed datasets. Results using unimputed models were materially similar so imputed models were used for greater statistical power. Patterns of missing data are shown in the supplementary material (Supplementary Table 4), these did not differ substantially between current smokers and not current smokers. Additionally, repeating the main analyses with longitudinal weights applied produced largely consistent results, and of note the relationship between baseline smoking and loneliness in subsequent waves was slightly stronger (Supplementary Table 5).

In addition to main analyses, we conducted the following sensitivity analyses: stratification by sex, and by age (above and below 65 years old), to identify if any relationships were more clearly present in one age group or sex than another; and using living alone as a covariate, rather than an outcome variable to assess if it modulated any relationships observed. Of note, the sensitivity analyses were exploratory in nature, and given the separation into smaller groups could be underpowered to detect differences, hence should be interpreted with caution. Sensitivity analyses were also conducted using complete case analysis. All our analyses were agreed a priori but we did not produce a formal protocol. All analyses were completed using Stata version 14.

### Role of the funding source

The funder had no role in the design or conduct of the study; the collection, management, analysis, or interpretation of the data; preparation, review, or approval of the manuscript; or in the decision to submit the manuscript for publication.

## Results

Table 1 shows baseline characteristics of the sample, and grouped according to their smoking status. For the total sample, mean age was 67 (SD 10), ranging from 52 to >90 years, 45% were male, 98% reported as white ethnicity, 31% were currently employed, 24% were classified as having depression, and 51% reported a medical condition. There were 1,329 (15·1%) current smokers and 7,451 (84·9%) not current smokers. Smokers compared with non-smokers, were slightly younger, with lower educational achievement levels, lower levels of net wealth, slightly higher rates of being in current employment, and more likely to be depressed.

**Table 1:** Baseline characteristics of the sample in relation to smoking status at baseline.

	Total	Not current smoker at baseline	Current smoker at baseline	p-value
	N=8,780	N=7,451	N=1,329	
**Social isolation and loneliness scores**				
Low social contact	4·99 (2·39)	4·93 (2·37)	5·35 (2·46)	**<0·001**
Social disengagement	5·32 (1·98)	5·16 (1·97)	6·24 (1·76)	**<0·001**
Living alone	26%	26%	31%	**<0·001**
Loneliness scale	4·12 (1·51)	4·07 (1·48)	4·38 (1·69)	**<0·001**
Baseline characteristics				
Age in years	67 (10)	68 (10)	64 (9)	**<0·001**
Male sex	45%	45%	46%	0·664
Ethnic minorities	2%	2%	2%	0·97
Educational attainment				**<0·001**
Degree	12%	13%	6%	
nvq3 A level/higher education	27%	28%	24%	
nvq2/gce o level	17%	17%	15%	
nvq1/cse or no qualification	44%	42%	54%	
**Wealth quintile (total net non-pension assets) (low to high)**				**<0·001**
1	20%	17%	36%	
2	20%	19%	23%	
3	20%	20%	17%	
4	19%	21%	13%	
5	20%	21%	10%	
Currently employed	31%	31%	34%	**0·008**
Depression	24%	22%	32%	**<0·001**
Mobility impairment	3%	4%	3%	0·130
Any medical diagnosis	51%	51%	51%	0·961

Notes: Data are presented as mean (SD) for continuous measures, and % for categorical measures. P values are for between group differences for current smokers vs non-smokers. Data in this table are unimputed.

**Figure 1:**
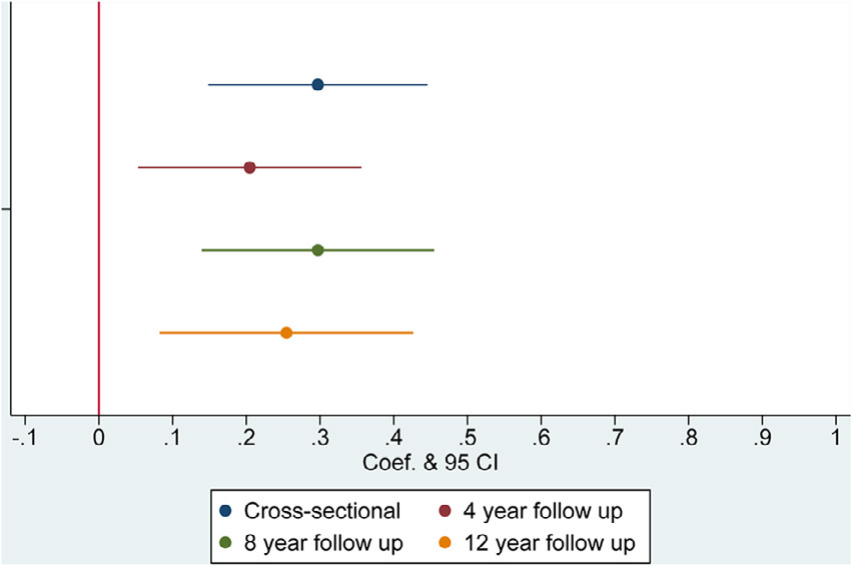
Smoking and low social contact.

**Figure 2:**
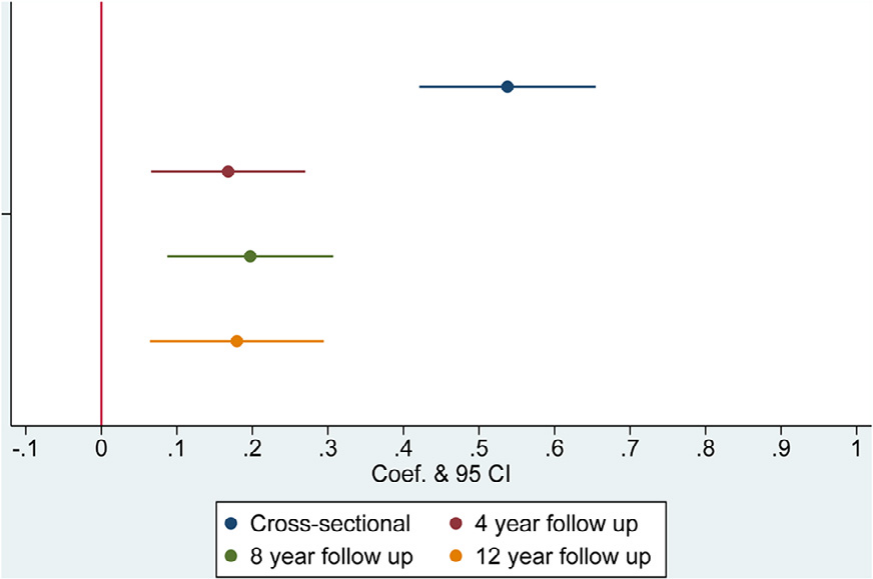
Smoking and Social Disengagement.

**Figure 3:**
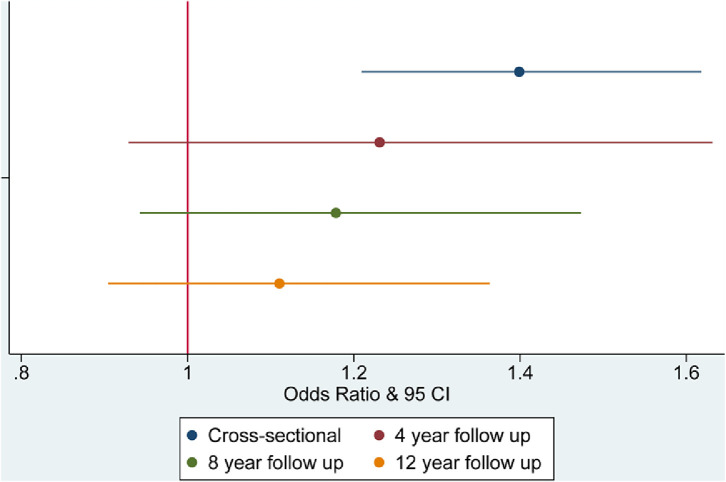
Smoking and Domestic Isolation.

**Figure 4:**
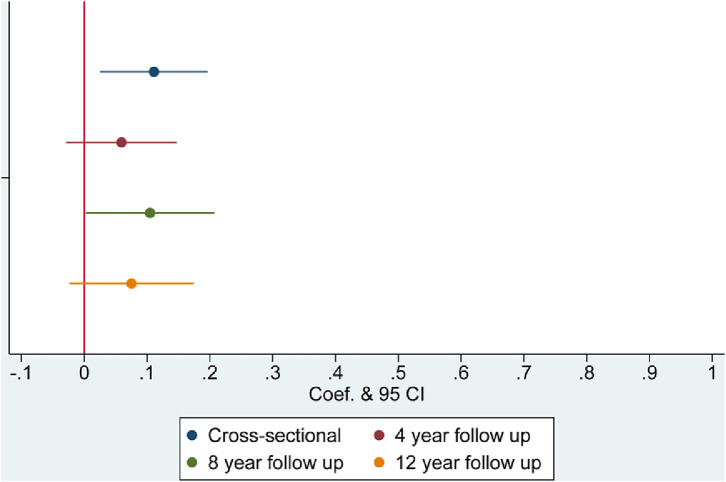
Smoking and Loneliness.

Results from regression models are presented in Table 2 and Figure 1, Figure 2, Figure 3, Figure 4, with coefficients corresponding to the difference in the dependent variable related to being a current smoker at baseline, compared with a being a non-smoker at baseline. At baseline, smokers were more likely to be lonely (coef.=0·111, 95% CI=0·025 to 0·196) and socially isolated than non-smokers, having less frequent social interactions with family and friends (coef.= 0·297, 95% CI=0·148 to 0·446), having less frequent engagement with community and cultural activities (coef.=0·534, 95% CI=0·421 to 0·654), and being more likely to live alone (OR=1·400, 95% CI=1·209 to 1·618) (Table 2). Longitudinally, current smoking at baseline was associated with larger reductions in social contact at 4, 8 and 12-year follow-up (coef.=0·205 to 0·254) and increased social disengagement (coef.=0·168 to 0·197). There was also evidence that smoking at baseline was associated with increased loneliness 4 years later (coef.=0·105, 95% CI=0·003 to 0·207) but this was not found at other timepoints. Smoking was not related to changes in cohabitation status in our sample. These findings were independent of all identified confounders.

**Table 2:** Multivariable adjusted associations between smoking status at baseline and social isolation and loneliness.

	Low social contact		Social disengagement		Domestic isolation		Loneliness	
	Coef (95% CI)	P	Coef (95% CI)	P	Odds Ratio (95% CI)	P	Coef (95% CI)	P
**Cross-sectional**								
Unadjusted	0·362 (0·212 to 0·512)	**<0·001**	0·876 (0·750 to 1·002)	**<0·001**	1·312 (1·155 to 1·490)	**<0·001**	0·323 (0·230 to 0·416)	**<0·001**
Model 1: Demographic covariates	0·324 (0·175 to 0·473)	**<0·001**	0·560 (0·442 to 0·678)	**<0·001**	1·452 (1·257 to 1·677)	**<0·001**	0·188 (0·096 to 0·281)	**<0·001**
Model 2: Demographic and Health related covariates	0·297 (0·148 to 0·446)	**<0·001**	0·534 (0·421 to 0·654)	**<0·001**	1·400 (1·209 to 1·618)	**<0·001**	0·111 (0·025 to 0·196)	**0·011**

**Longitudinal: Wave 2 to Wave 4**								
Adjusted: Model 2 & baseline level of outcome measure	0·205 (0·053 to 0·356)	**0·008**	0·168 (0·066 to 0·270)	**0·001**	1·231 (0·929 to 1·631)	0·148	0·059 (-0·029 to 0·147)	0·179
**Longitudinal: Wave 2 to Wave 6**								
Adjusted: Model 2 & baseline level of outcome measure	0·297 (0·140 to 0·455)	**<0·001**	0·197 (0·087 to 0·307)	**<0·001**	1·178 (0·943 to 1·473)	0·149	0·105 (0·003 to 0·207)	**0·045**
**Longitudinal Wave 2 to Wave 8**								
Adjusted: Model 2 & baseline level of outcome measure	0·254 (0·083 to 0·426)	**0·004**	0·179 (0·064 to 0·294)	**0·002**	1·110 (0·904 to 1·364)	0·317	0·075 (-0·024 to 0·174)	0·136

Subgroup analysis by sex showed broadly equivalent results for male and female for measures of social isolation, except for slight attenuation of the associations of smoking with low social contact in men at 4- and 12-year follow-ups. Additionally, the relationship between smoking and loneliness was largely maintained in males, but not in females, when analysed separately (Supplementary Table 1). Subgroup analysis by age confirmed the relationships of smoking with social isolation variables found in the main analyses in the <65 age group, but these relationships were attenuated in the ≥65years old group. Cross-sectional and longitudinal associations between smoking and loneliness were notably stronger in the <65 age group than in the ≥65year old group (Supplementary Table 2). As stated above, caution is required when interpreting the subgroup analyses given their exploratory nature, and potential to be statistically underpowered.

Results were consistent in analyses where living alone was used as an additional covariate in models for low social contact and social disengagement (Supplementary Table 3). The cross-sectional relationship of smoking with loneliness was attenuated, however the longitudinal relationships from wave 2 to wave 6 remained statistically significant.

## Discussion

### Principle findings

This study assessed the relationship of smoking with current, and future levels of social isolation and loneliness. We analysed data covering a 12-year follow-up period in a nationally representative sample of adults aged 52 and over in England. We found no evidence to support the idea that smoking might be prosocial, which appears therefore to be a misconception; indeed, our results suggest smokers have higher levels of social isolation and loneliness over time, with less contact with friends and family members outside the household than non-smokers. Subgroup analysis suggested slight variations related to age and sex. Smoking was cross-sectionally associated with an increased chance of living alone, but not longitudinally related to changes in domestic isolation. The direction of the relationship between these variables is unclear. People might be less enthusiastic about living with a smoker, people may be more likely to smoke if living alone, or a bidirectional multifactorial relationship might explain the association seen. Given the strong relationships between social isolation, loneliness, and negative health outcomes,^20^ the results presented here suggest that smoking may not only contribute to poor health and premature mortality via well-known direct and indirect tobacco-related pathways, but also via exacerbating the effects of social factors on morbidity and mortality.

#### Comparison with other studies

Our cross-sectional results corroborate previous studies of middle aged and older adults suggesting smokers are more likely to be socially isolated,^7^, ^8^, ^9^ and (albeit to a slightly lesser extent) lonely,^7^^,^^21^ compared with non-smokers. Longitudinal research on this topic is more limited, although social network analysis has shown that smokers can become increasingly marginalised in society over time, in keeping with our findings.^22^

Considering potential mechanisms for the relationships observed, several small studies have explored ways in which social norms and legislation regarding smoking could impact social isolation and loneliness in smokers. For example, a longitudinal qualitative study of 32 smokers (age categories 18-30 to >60 years) explored social and behavioural impacts of English smoke-free legislation (SFL).^12^ The study found SFL had negative social impacts on participants, particularly those aged over 60, which the study suggested might increase social isolation, though of note most younger adult smokers felt relatively unaffected by the legislation. Some participants reported opting to stay at home more, where they could smoke, rather than going to social or public spaces where smoking was prohibited. This study suggests the relationship between smoking, social isolation and loneliness may change over the life course. Younger adults are likely to experience lower levels of smoking related diseases impacting mobility and physical capacity to socialise. However, differences in smoking related social norms are also likely to be relevant, as starting smoking, and quit attempts, in younger people are highly influenced by social interactions and context.^2^^,^^3^ Currently, research on these topics over the life course is lacking. Another potential explanatory mechanism relates to smoking stigma from others, and self,^23^ in part due to the de-normalisation of smoking in various contexts, which is relevant to adults of all age groups.^6^^,^^24^^,^^25^ De-normalisation of smoking, stigma, and SFL are intricately linked components of broader societal changes related to smoking. Each of these factors is likely to have contributed to the relationships found in our study, with the relative contribution of each dependent on the circumstances and experiences of individuals. These social changes have become prominent in recent years and remain dynamic. As such, the way in which they influence the relationships described will continue to develop, in what way however is difficult to say. An additional consideration is that smokers who are married are more likely to quit, so may well have been non-smokers by the baseline.^26^ Furthermore, given the interconnectedness of smoking in social networks,^22^ smoking related morbidity and mortality are likely to impact the social contacts of smokers as well as themselves, compounding impacts. Such studies support the proposed explanatory mechanisms for our findings which demonstrates these relationships in cross-sectional and longitudinal analyses of a nationally representative sample.

Previous research has largely focused on social isolation and loneliness leading to increased smoking^7^, ^8^, ^9^, while our findings suggests smoking can also lead to increased levels of social isolation and loneliness. Hence, the causal pathways between these factors are likely bidirectional, suggesting a vicious cycle of smoking, social isolation, and loneliness. These findings have important policy implications. Firstly, they further strengthen the justification for anti-smoking legislation, through identifying psychosocial, in addition to physical, harms related to smoking. Secondly, they emphasise the overlapping and often interdependent nature of biological, psychological and social aspects of health, and in doing so, should prompt unified policy approaches to address such issues.

#### Strengths and Limitations of the study

Our study builds on previous research in various ways. Firstly, previous research largely focused on hypotheses exploring how social isolation and loneliness could lead to increased levels of smoking, rather than the direction explored here. To our knowledge, this is the first study to assess the longitudinal relationship between baseline smoking and levels of future social isolation and loneliness in a representative cohort. Secondly, by using three measures of social isolation, our findings enable a more nuanced understanding of how smoking is related to different aspects of social isolation. Thirdly, assessing these relationships over a 12-year follow-up period enables associations to be demonstrated that would be difficult to identify over shorter periods. Fourthly, by embedding the analysis in a well-established multidisciplinary longitudinal study, we were able to take advantage of the wide set of health, economic, and social variables available, properly controlling for potential confounders.

Certain limitations and considerations should be mentioned. Firstly, as an observational study, causality cannot be assumed, and unmeasured confounding factors may have contributed to the results. However, there is a strong argument supporting potential causal mechanisms including cumulative smoking related illness, changing social norms regarding smoking, and antismoking legislature in public spaces. Additionally, the results were robust to multiple sensitivity analyses and in keeping with related research in this area. Secondly, data used were from adults aged 50 years old and above, therefore the extent these findings can be generalised to other age groups is not clear. Thirdly, the cross-cultural generalisability of our findings is unclear. Further research on this topic, particularly in countries where cultural activities that involve smoking are more common (such as “sheesha” or “mu'assel”) would be valuable, with further exploration of sex and age differences related to smoking in other cultures. However, having used a nationally representative sample from England, the generalisability of these findings within this context are reasonably robust. Fourthly, detailed information regarding other types of tobacco use, vaping/e-cigarettes, and more accurate quantification of smoking (for example ‘pack years’), may have provided more information on the relationships observed, but these data were not available in the present study. Though of note, other research suggests e-cigarette use was extremely low at the time of the first two waves used in this study.^27^ Fifthly, our sample included a slightly higher proportion of female than male smokers, while national data suggested slightly higher rates of male than female smokers in this age group at the time.^28^ Overall, the difference is small, and at least partly due to our overall sample being 55% female, but still worthy of note. Finally, missing data increases into later waves (Supplementary Table 4), and although we have taken a clear, logical, and robust approach to its handling, it would be prudent to apply a degree of caution in the interpretation of 12-year follow up findings.

### Conclusions and study implications

In conclusion, this study suggests that smoking leads to increased social isolation and loneliness in older adults. These associations were more prominent for social isolation than loneliness, especially in women, and differed slightly by age group, with smoking and loneliness more strongly associated in people aged 52-65, than those aged 65 and above.

This study raises various future research questions. Firstly, the longitudinal relationship between quitting smoking, social isolation and loneliness requires further exploration, including the relationship between changing smoking behaviours and social activities, and whether changing social pressures are associated with decisions to quit. Of note, elements of these topics have been touched upon by a recent Cochrane systematic review,^29^ and other research,^30^ which suggests that smoking cessation is related to improvements in anxiety, depression, social well-being, and social isolation.^29^ Secondly, it would be useful to assess the cross-cultural consistency of our findings with research on this topic in other settings, which is likely to modulate the relationships found.^12^^,^^21^ Thirdly, future research should explore in greater detail potential causal pathways underlying the relationships described here, particularly given the increased rates of smoking in already marginalised and disadvantaged groups of society, to help mitigate impacts. Fourthly, these findings highlight the possibility that increasing social isolation and loneliness secondary to smoking could be a contributory factor to pathways between smoking and its subsequent increased morbidity and mortality; an area in which further research is warranted. Additionally, research exploring how these relationships differ by extent of life-long tobacco exposure (e.g. pack years) and among vaping/e-cigarette smokers are all important future research topics. Similarly, it would be interesting to explore whether heavier or more frequent smoking was related to larger changes in social isolation and loneliness, than occasional or ‘lighter’ smoking. An overarching consideration is that smoking habits within societies change overtime, therefor research exploring how these relationships might also change overtime would be valuable.

Adding to other research on the health impacts of smoking, our results suggest that smoking is potentially detrimental to aspects of psychosocial health. These findings emphasise the intersection of two major public health priorities which requires further attention. In policy terms it provides a further basis to increase efforts to achieve Smokefree society.^31^ Preventing smoking uptake is important but attention to strategies that will support older smokers to quit is also essential, in particular targeting less affluent groups, at risk occupations, and people with mental health problems where smoking rates are disproportionately high. Prosocial and cost-effective, yet chronically underfunded, smoking cessation services deserve investment.^32^ Although causality cannot be assumed, and further research is warranted, the idea held by some that smoking might be prosocial appears to be a misconception, with serious implications for health and wellbeing throughout the lifespan.

## Contributors

KEJP, FB, DF, and NSH had the original idea for the study. All authors (KEJP, FB, MIP, JB, AS, NSH, DF) contributed to the design of the study. KEJP, with contributions from FB and AS, carried out the analyses. KEJP drafted the manuscript. All authors (KEJP, FB, MIP, JB, AS, NSH, DF) critically appraised the manuscript and approved it for submission. KEJP, FB, and AS had full access to the data and can take responsibility for the integrity of the data and the accuracy of the data analysis. KEJP, FB, and AS act as guarantors. The corresponding author attests that all listed authors (KEJP, FB, MIP, JB, AS, NSH, DF) meet authorship criteria and that no others meeting the criteria have been omitted.

## Declaration of interest

All authors have completed the ICMJE uniform disclosure form and declare: support from the National Institute of Ageing, a consortium of UK government departments coordinated by the Economic and Social Research Council; Imperial College London; and the Wellcome Trust for the submitted work; no financial relationships with any organisations that might have an interest in the submitted work in the previous three years, no other relationships or activities that could appear to have influenced the submitted work.

## Appendix Supplementary materials

Image, application 1
