# Supplementary material for: Relationship of smoking with current and future social isolation and loneliness: 12-year follow-up of older adults in England
Source: Lancet Reg Health Eur. 2022 Jan 2;14:100302. doi: 10.1016/j.lanepe.2021.100302 (PMC8743222; doi:10.1016/j.lanepe.2021.100302)
Supplement: Supplementary file 1 [file mmc1.docx]

# **Supplementary File**

# **Title:** **Is smoking social? The relationship of smoking with current and future social isolation and loneliness: 12-year follow-up of 8,780 older adults in England**

Keir EJ Philip^1,2,3*^ (0000-0001-9614-3580), Feifei Bu^4^ (0000-0003-2060-3768), Michael I Polkey^1,3^ (0000-0003-1243-8571), Jamie Brown^4^ (0000-0002-2797-5428), Andrew Steptoe^5^ (0000-0001-7808-4943) Nicholas S Hopkinson^1,2,3**^ (0000-0003-3235-0454), Daisy Fancourt^5**^ (0000-0002-6952-334X)

1. National Heart and Lung Institute, Imperial College London, London, United Kingdom
2. NIHR Imperial Biomedical Research Centre, London, United Kingdom
3. Respiratory Medicine, Royal Brompton Business Group, Guys and St Thomas’ NHS Foundation Trust, London, United Kingdom
4. Tobacco and Alcohol Research Group, University College London, United Kingdom
5. Department of Behavioural Science and Health, University College London, London, United Kingdom

*Corresponding Author: Keir EJ Philip, email: k.philip@imperial.ac.uk

**Contents:**

| Page |  |
| --- | --- |
| 1 | Supplementary Table 1: Results from regression models showing the relationship of smoking, with social isolation and loneliness, split by sex |
| 2 | Supplementary Table 2: Results from regression models showing the relationship between smoking, and social isolation and loneliness, split by age (above and below 65 years old) |
| 3 | Supplementary Table 3: Results from regression models showing the relationship between smoking, and social isolation and loneliness, with domestic isolation as a covariate in addition to demographic and health related covariates |
| 4 | Supplementary Table 4: Pattern of missing data prior to imputations |
| 4 | Supplementary Table 5: Results from regression models showing the relationship between smoking, and social isolation and loneliness over time, with longitudinal weights applied in addition to demographic and health related covariates |

**Supplementary Tables: Sensitivity analyses**

**Supplementary table 1: Results from regression models showing the relationship of smoking, with social isolation and loneliness, split by sex**

|  | Low social contact | | Social disengagement | | Domestic isolation | | Loneliness | |
| --- | --- | --- | --- | --- | --- | --- | --- | --- |
|  | Coef (95% CI) | P | Coef (95% CI) | P | Odds Ratio (95% CI) | P | Coef (95% CI) | P |
| **MEN (n=3949)** | | | | | | | | |
| Cross-sectional | 0·263 (0·050 to 0·477) | **0·016** | 0·386 (0·209 to 0·562) | **<0·001** | 1·485 (1·184 to 1·862) | **0·001** | 0·125 (0·006 to 0·245) | **0·039** |
| Longitudinal: Wave 2 to Wave 4 | 0·151 (-0·082 to 0·384) | 0·204 | 0·151 (0·005 to 0·297) | **0·042** | 1·451 (0·914 to 2·303) | 0·114 | 0·197 (-0·033 to 0·227) | 0·144 |
| Longitudinal: Wave 2 to Wave 6 | 0·304 (0·049 to 0·560) | **0·020** | 0·217 (0·057 to 0·378) | **0·008** | 1·196 (0·806 to 1·774) | 0·374 | 0·136 (-0·060 to 0·223) | 0·076 |
| Longitudinal: Wave 2 to Wave 8 | 0·205 (-0·054 to 0·464) | 0·120 | 0·183 (0·009 to 0·356) | **0·039** | 1·230 (0·887 to 1·708) | 0·214 | 0·081 (-0·047 to 0·242) | 0·258 |
| **WOMEN (n=4831)** | | | | | | | | |
| Cross-sectional | 0·332 (0·128 to 0·537) | **0·001** | 0·664 (0·508 to 0·820) | **<0·001** | 1·329 (1·099 to 1·607) | **0·003** | 0·102 (-0·020 to 0·223) | 0·100 |
| Longitudinal: Wave 2 to Wave 4 | 0·247 (0·039 to 0·454) | **0·020** | 0·181 (0·048 to 0·315) | **0·008** | 1·116 (0·783 to 1·592) | 0·544 | 0·024 (-0·100 to 0·148) | 0·700 |
| Longitudinal: Wave 2 to Wave 6 | 0·295 (0·082 to 0·508) | **0·007** | 0·185 (0·041 to 0·328) | **0·012** | 1·166 (0·874 to 1·556) | 0·296 | 0·076 (-0·057 to 0·209) | 0·260 |
| Longitudinal Wave 2 to Wave 8 | 0·300 (0·067 to 0·532) | **0·012** | 0·178 (0·021 to 0·335) | **0·026** | 1·037 (0·792 to 1·359) | 0·790 | 0·067 (-0·068 to 0·202) | 0·331 |

Notes: All results are adjusted for Model 1 (demographic) and Model 2 (health-related) covariates. Longitudinal analyses additionally control for the baseline value of the variable under investigation.

**Supplementary table 2: Results from regression models showing the relationship between smoking, and social isolation and loneliness, split by age (above and below 65 years old)**

|  | Low social contact | | Social disengagement | | Domestic isolation | | Loneliness | |
| --- | --- | --- | --- | --- | --- | --- | --- | --- |
|  | Coef (95% CI) | P | Coef (95% CI) | P | Odds Ratio (95% CI) | P | Coef (95% CI) | P |
| **65 years old or older (n=4706)** | | | | | | | | |
| Cross-sectional | 0·352 (0·120 to 0·585) | **0·003** | 0·491 (0·304 to 0·678) | **<0·001** | 1·402 (1·143 to 1·719) | **0·001** | 0·082 (-0·054 to 0·218) | 0·236 |
| Longitudinal: Wave 2 to Wave 4 | 0·174 (-0·066 to 0·414) | 0·155 | 0·134 (-0·013 to 0·282) | 0·075 | 1·395 (0·935 to 2·082) | 0·102 | 0·040 (-0·108 to 0·188) | 0·593 |
| Longitudinal: Wave 2 to Wave 6 | 0·215 (-0·036 to 0·466) | 0·093 | 0·153 (-0·022 to 0·328) | 0·086 | 1·202 (0·853 to 1·694) | 0·292 | 0·035 (-0·132 to 0·202) | 0·680 |
| Longitudinal Wave 2 to Wave 8 | 0·199 (-0·066 to 0·465) | 0·141 | 0·151 (-0·027 to 0·330) | 0·096 | 1·091 (0·805 to 1·479) | 0·573 | 0·022 (-0·137 to 0·181) | 0·788 |
|  | | | | | | | | |
| **Under 65 years old (n=4074)** | | | | | | | | |
| Cross-sectional | 0·238 (0·041 to 0·436) | **0·018** | 0·571 (0·420 to 0·722) | **<0·001** | 1·370 (1·110 to 1·690) | **0·003** | 0·124 (0·015 to 0·233) | **0·026** |
| Longitudinal: Wave 2 to Wave 4 | 0·226 (0·021 to 0·431) | **0·031** | 0·196 (0·069 to 0·323) | **0·003** | 1·089 (0·745 to 1·592) | 0·659 | 0·072 (-0·049 to 0·193) | 0·241 |
| Longitudinal: Wave 2 to Wave 6 | 0·359 (0·158 to 0·561) | **0·001** | 0·225 (0·088 to 0·362) | **0·001** | 1·149 (0·854 to 1·547) | 0·360 | 0·155 (0·030 to 0·280) | **0·016** |
| Longitudinal Wave 2 to Wave 8 | 0·294 (0·089 to 0·500) | **0·005** | 0·194 (0·049 to 0·340) | **0·009** | 1·110 (0·842 to 1·465) | 0·458 | 0·107 (-0·015 to 0·228) | 0·086 |

Notes: All results are adjusted for Model 1 (demographic) and Model 2 (health-related) covariates. Longitudinal analyses additionally control for the baseline value of the variable under investigation.

**Supplementary table 3: Results from regression models showing the relationship between smoking, and social isolation and loneliness, with domestic isolation as a covariate in addition to demographic and health related covariates**

|  | Low social contact | | Social disengagement | | Loneliness | |
| --- | --- | --- | --- | --- | --- | --- |
|  | Coef (95% CI) | P | Coef (95% CI) | P | Coef (95% CI) | P |
| Cross-sectional | 0·273 (0·125 to 0·422) | **<0·001** | 0·552 (0·436 to 0·669) | **<0·001** | 0·069 (-0·015 to 0·153) | 0·106 |
| Longitudinal: Wave 2 to Wave 4 | 0·196 (0·044 to 0·348) | **0·012** | 0·170 (0·067 to 0·272) | **0·001** | 0·055 (-0·033 to 0·143) | 0·223 |
| Longitudinal: Wave 2 to Wave 6 | 0·297 (0·139 to 0·456) | **<0·001** | 0·199 (0·089 to 0·309) | **<0·001** | 0·109 (0·006 to 0·212) | **0·037** |
| Longitudinal: Wave 2 to Wave 8 | 0·252 (0·080 to 0·424) | **0·004** | 0·177 (0·061 to 0·293) | **0·003** | 0·083 (-0·015 to 0·182) | 0·097 |

Notes: All results are adjusted for domestic isolation, and Model 1 (demographic) and Model 2 (health-related) covariates. Longitudinal analyses additionally control for the baseline value of the variable under investigation.

**Supplementary Table 4: Pattern of missing data prior to imputations**

| Variable | Complete | Missing | Percentage Missing |
| --- | --- | --- | --- |
| Age | 8780 | 0 | 0 |
| Educational attainment | 8770 | 10 | 0 |
| Wealth | 8661 | 119 | 1 |
| Gender | 8780 | 0 | 0 |
| Ethnicity | 8780 | 0 | 0 |
| Current employment | 8778 | 2 | 0 |
| Medical diagnoses | 8776 | 4 | 0 |
| Depression | 8667 | 113 | 1 |
| Mobility impairment | 8603 | 177 | 2 |
| Low social contact (wave 2) | 6846 | 1934 | 22 |
| Low social contact (wave 4) | 5204 | 3576 | 41 |
| Low social contact (wave 6) | 4348 | 4432 | 51 |
| Low social contact (wave 8) | 3998 | 4782 | 54 |
| Social disengagement (wave 2) | 6033 | 2747 | 31 |
| Social disengagement (wave 4) | 4884 | 3896 | 44 |
| Social disengagement (wave 6) | 4309 | 4471 | 51 |
| Social disengagement (wave 8) | 3089 | 5691 | 65 |
| Domestic isolation (wave 2) | 8780 | 0 | 0 |
| Domestic isolation (wave 4) | 6260 | 2520 | 29 |
| Domestic isolation (wave 6) | 5316 | 3464 | 39 |
| Domestic isolation (wave 8) | 3998 | 4782 | 54 |
| Loneliness (wave 2) | 7587 | 1193 | 14 |
| Loneliness (wave 4) | 5284 | 3496 | 40 |
| Loneliness (wave 6) | 4552 | 4228 | 48 |
| Loneliness (wave 8) | 3412 | 5368 | 61 |

**Supplementary table 5: Results from regression models showing the relationship between smoking, and social isolation and loneliness over time, with longitudinal weights applied in addition to demographic and health related covariates**

|  | Low social contact | | Social disengagement | | Domestic isolation | | Loneliness | |
| --- | --- | --- | --- | --- | --- | --- | --- | --- |
|  | Coef (95% CI) | P | Coef (95% CI) | P | Odds Ratio (95% CI) | P | Coef (95% CI) | P |
| Longitudinal: Wave 2 to Wave 4 | 0·255 (0.071 to 0.439) | **0·007** | 0·212 (0·100 to 0·325) | **<0·001** | 1.198 (0.839 to 1.710) | 0.321 | 0·104 (-0·013 to 0·221) | 0·081 |
| Longitudinal: Wave 2 to Wave 6 | 0·413 (0·199 to 0·628) | **<0·001** | 0·312 (0·175 to 0·448) | **<0·001** | 1.124 (0.821 to 1.539) | 0.465 | 0·158 (0·018 to 0·298) | **0.027** |
| Longitudinal: Wave 2 to Wave 8 | 0·289 (0·048 to 0·532) | **0·019** | 0·252 (0·076 to 0·428) | **0·005** | 1.132 (0.821 to 1.561) | 0.450 | 0·165 (0·013 to 0·317) | **0.034** |
